# Memory, social cognition, and beyond: Neuropsychological markers differentiating Alzheimer's disease and frontotemporal dementia

**DOI:** 10.1177/13872877261480113

**Published:** 2026-09-02

**Authors:** Jana Marie Freitag, Franz Felix Konen, Johannes Heck, Kirsten Jahn, Stefanie Groba, Martin Klietz, Thomas Skripuletz, Florian Wegner, Martin Schulze Westhoff, Sebastian Schröder

**Affiliations:** 1 Department of Psychiatry, Social Psychiatry and Psychotherapy, 9177 Hannover Medical School, Hannover, Germany; 2 PRACTIS Clinician Scientist Program, 9177 Hannover Medical School, Hannover, Germany; 3 Department of Neurology, 9177 Hannover Medical School, Hannover, Germany; 4 Institute for General Practice and Palliative Care, 9177 Hannover Medical School, Hannover, Germany; 5 Laboratory of Molecular Neurosciences, Department of Psychiatry, Social Psychiatry and Psychotherapy, 9177 Hannover Medical School, Hannover, Germany

**Keywords:** Alzheimer's disease, differential diagnosis, episodic memory, frontotemporal dementia, neuropsychological assessment, social cognition

## Abstract

**Background:**

Distinguishing Alzheimer's disease (AD) from frontotemporal dementia (FTD) remains a major clinical challenge, particularly in early disease stages due to overlapping symptoms. Although neuropsychological assessment is central to diagnosis, brief cognitive screening instruments often emphasize episodic memory, whereas comprehensive neuropsychological assessment encompasses multiple cognitive domains. Nevertheless, social cognition and other relevant functions may remain underrepresented in routine clinical assessment.

**Objective:**

To identify neuropsychological domains and test procedures that reliably differentiate AD from FTD and support differential diagnosis.

**Methods:**

A systematic PubMed literature search was conducted using predefined inclusion and exclusion criteria. Original studies directly comparing neuropsychological performance in patients with AD and FTD were included. Owing to methodological heterogeneity, findings were synthesized narratively.

**Results:**

A total of 322 records were identified, of which 80 studies met the inclusion criteria. A clear domain-specific pattern emerged. Episodic memory impairment, particularly delayed recall deficits, consistently distinguished AD from FTD, with poorer performance in AD. In contrast, deficits in social cognition, including theory of mind and emotion recognition, were more pronounced in FTD and were often detectable early in the disease course. Executive functions and language showed heterogeneous findings, with discriminative value depending on specific subdomains and FTD subtypes. Visuospatial functions and attention provided supportive but less consistent differentiation, while global screening instruments showed limited diagnostic specificity.

**Conclusions:**

Differentiation between AD and FTD should not rely on single cognitive domains. The most clinically meaningful distinction is achieved through a multidimensional assessment integrating episodic memory, social cognition, and selected executive and behavioral measures.

## Introduction

Alzheimer's disease (AD) and frontotemporal dementia (FTD) are two of the most common neurodegenerative disorders and are major causes of cognitive decline.^1–3^ Accurate and early differentiation between these conditions is essential, as it has important implications for clinical management, therapy, prognosis and patient counselling. However, particularly in the early stages of the disease, overlapping clinical features can make a reliable differential diagnosis difficult.^4^

AD is characterized by early impairments in episodic memory, reflecting medial temporal lobe involvement.^5^ In contrast, FTD and especially the behavioral variant (bvFTD) is primarily associated with changes in personality, behavior, and social cognition.^6^ Nevertheless, this conventional distinction is often blurred. Patients with FTD may also exhibit early impairments in memory performance, although these are typically less pronounced and may reflect executive or retrieval deficits rather than primary episodic memory dysfunction.^6^ Meanwhile, atypical variants of AD can manifest with behavioral and executive dysfunction, further complicating diagnostic differentiation.^1,4,7–9^

Neuropsychological assessment plays a central role in diagnosing patients with suspected neurodegenerative disorders.^10^ However, although comprehensive neuropsychological assessment evaluates multiple cognitive domains, brief cognitive screening instruments frequently emphasize episodic memory, potentially limiting the detection of characteristic cognitive profiles such as those observed in bvFTD.^11,12^ Recent studies highlight the importance of additional domains, such as social cognition and executive functioning, for differential diagnosis. However, executive functioning represents a heterogeneous construct comprising partially overlapping cognitive processes, including inhibition, cognitive flexibility, working memory, and verbal fluency.^13^ Furthermore, the conceptual definitions and subdomain classifications of executive functions vary across neuropsychological frameworks and research traditions. This variation may contribute to inconsistencies across studies and complicate direct comparisons between findings. Deficits in social cognition, in particular, were shown to distinguish bvFTD from AD independently of memory performance.^14,15^

Numerous individual studies have investigated neuropsychological differences between AD and FTD using a wide range of cognitive tests and study populations.^16,17^ Some consistent findings have emerged, such as impaired episodic memory in AD and deficits in social cognition in FTD.^16,18,19^ However, results for other domains, especially executive functions, remain heterogeneous.^17,20^ Furthermore, variability in study design, patient cohorts and the neuropsychological instruments used, yields in limited comparability across studies.

Despite this growing body of literature, a comprehensive synthesis that systematically integrates findings across multiple cognitive domains is still lacking.^1^ Such synthesis is crucial in order to identify robust, clinically applicable markers for differential diagnosis, and to develop optimized neuropsychological test batteries.

The aim of this systematic review is therefore to identify cognitive domains and neuropsychological test procedures reliably differentiating AD from FTD, and to evaluate their clinical relevance.

## Methods

### Search strategy

A systematic literature search was conducted in PubMed on the 19^th^ of January 2026 to identify studies investigating neuropsychological differences between AD and FTD. The search strategy included terms related to AD and FTD, neuropsychological assessment, cognitive functioning, executive function, and social cognition, as well as terms related to differentiation and comparison between the two disorders.

The following search strategy was applied:

(“frontotemporal dementia"[Title/Abstract] OR bvFTD[Title/Abstract])

AND

(“Alzheimer disease"[Title/Abstract] OR Alzheimer*[Title/Abstract])

AND

(neuropsycholog*[Title/Abstract] OR cognition[Title/Abstract] OR “cognitive profile"[Title/Abstract] OR “executive function*"[Title/Abstract] OR “social cognition"[Title/Abstract])

AND

(differenti*[Title/Abstract] OR distinguish*[Title/Abstract] OR comparison[Title/Abstract])

No restrictions regarding publication period were applied. Only English-language studies were included.

### Eligibility criteria

Original studies directly comparing quantitative neuropsychological test performance of patients with AD and FTD were eligible for inclusion. Reviews, editorials, case reports, animal studies, imaging- or biomarker-only studies and studies without a direct comparison between AD and FTD were excluded.

### Study selection and data extraction

Study selection was conducted according to the PRISMA 2020 guidelines.^21^ After title and abstract screening, potentially relevant studies underwent full-text assessment. The study selection process is illustrated in Figure 1. Titles and abstracts were screened for eligibility by a single reviewer (J.M.F.), followed by full-text assessment of potentially eligible studies. No independent duplicate screening or data extraction was performed. Data extraction was also performed by the same reviewer using a standardized extraction form. Methodological quality was assessed using the Joanna Briggs Institute (JBI) Critical Appraisal Checklist. Uncertainties regarding the risk-of-bias assessment were resolved through discussion among the authors until consensus was reached.

**Figure 1.**
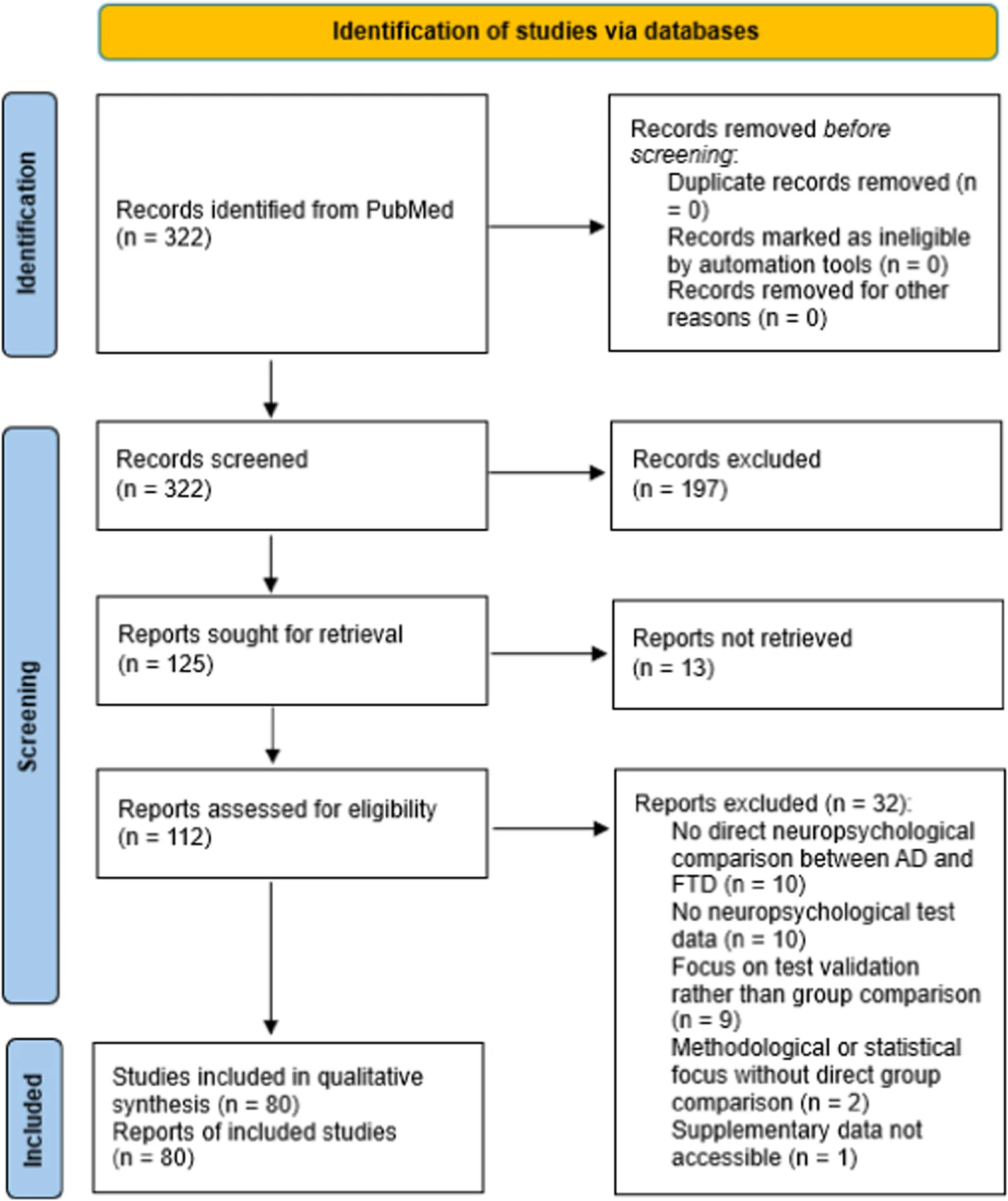
PRISMA 2020 flow diagram of study selection process.

For each included study, the following data were extracted: authors; publication year; study design; sample size; FTD subtype; assessed cognitive domains; applied neuropsychological tests; and reported group differences. The diagnostic definitions of AD used in the included studies were extracted and are summarized in Table 1.

**Table 1.** **Detailed characteristics of included studies (alphabetical)** (AD diagnostic criteria were categorized as follows: probable AD (NINCDS-ADRDA), probable AD (NIA-AA/McKhann 2011), biomarker-supported AD (clinical diagnosis supported by AD biomarkers), biomarker-confirmed AD (AD biomarkers required for diagnosis), and neuropathologically confirmed AD.).

Author	Year	AD diagnostic criteria	Study design	Sample size (AD/FTD)	FTD subtype	Primary domain	Tests	Main findings	Risk of bias
Amaral-Carvalho et al. ^22^	2023	Probable AD (NIA-AA/McKhann 2011)	Cross-sectional	102 / 37	bvFTD	Memory / Executive	ACE-R	AD worse memory	Moderate
Bahia and Viana ^23^	2009	Probable AD (NINCDS-ADRDA)	Cross-sectional, Retrospective	12 / 12	Not specified	Memory / Executive	DS-B, Category fluency	No difference	High
Bahia et al. ^24^	2018	Probable AD (NIA-AA/McKhann 2011)	Cross-sectional	20 / 18	bvFTD	Memory / Executive	RAVLT, Stroop, Category fluency	AD worse memory	Moderate
Baldock et al. ^25^	2016	Biomarker-supported AD	Cross-sectional	102 / 27	bvFTD, lvFTD	Memory / Language	HVLT-R, BVMT-R, BNT	AD worse memory	Moderate
Barrows et al. ^26^	2015	Probable AD (NINCDS-ADRDA)	Cross-sectional	16 / 15	bvFTD, eAD	Memory / Executive	WMS, DS-F, DS-B	AD worse memory	Moderate
Bertoux et al. ^18^	2013	Probable AD (NINCDS-ADRDA)	Cross-sectional	20 / 20	bvFTD	Social cognition / Executive	FAB, Go/No-Go, Mini-SEA	FTD worse social cognition	Moderate
Bertoux et al. ^27^	2014	Biomarker-confirmed AD	Cross-sectional	65 / 56	bvFTD	Memory	FCSRT	Mixed results	Moderate
Bertoux et al. ^14^	2016	Biomarker-supported AD	Cross-sectional	28 / 38	amnestic bvFTD, non-amnestic bvFTD (n = 19 each)	Memory / Social cognition	FCSRT, DS-F, DS-B	AD worse memory	Moderate
Braaten et al. ^28^	2006	Probable AD (NINCDS-ADRDA)	Cross-sectional, Retrospective	30 / 20	Not specified	Memory / Executive	WMS, TMT-B, Stroop	AD worse memory	Moderate
Buhl et al. ^29^	2013	Probable AD (NINCDS-ADRDA)	Cross-sectional	10 / 11	bvFTD	Social cognition / Executive	HSCT, FAB, ToM	FTD worse social cognition	High
Carey et al. ^30^	2008	Probable AD (NINCDS-ADRDA)	Cross-sectional	30 / 44	Not specified	Executive	D-KEFS	No difference	Moderate
Castiglioni et al. ^31^	2006	Probable AD (NINCDS-ADRDA)	Cross-sectional	85 / 33	bvFTD	Memory / Executive	ROCF, TMT-A, TMT-B	AD worse memory	Moderate
Custodio et al. ^32^	2016	Probable AD (NINCDS-ADRDA)	Cross-sectional	35 / 34	bvFTD	Executive	TMT-B, WCST, FAS	Mixed results	Moderate
Custodio et al. ^33^	2021	Probable AD (NIA-AA/McKhann 2011)	Cross-sectional	33 / 18	bvFTD	Social cognition / Executive	IFS, Mini-SEA, SEA	FTD worse social cognition	Moderate
De Icaza Valenzuela et al. ^34^	2021	Biomarker-supported AD	Cross-sectional, Retrospective	32 / 16	bvFTD	Memory / Social cognition	ACE-III	No difference	Moderate
Diehl et al. ^35^	2005	Probable AD (NINCDS-ADRDA)	Cross-sectional, Retrospective	69 / 51	bvFTD, svPPA	Memory / Executive	Category fluency, BNT	FTD worse language	Moderate
Dodich et al. ^36^	2018	Biomarker-supported AD	Cross-sectional, Retrospective	47 / 48	bvFTD	Memory / Social cognition	RAVLT, ROCF, DS-F	FTD worse social cognition	Moderate
Dodich et al. ^37^	2021	Probable AD (NIA-AA/McKhann 2011)	Cross-sectional	26 / 32	bvFTD	Social cognition	Ek-60F, SET	No difference	Moderate
Dominguez et al. ^38^	2022	Biomarker-confirmed AD	Cross-sectional	16 / 67	bvFTD	Memory / Executive	ROCF, DS-F, DS-B	AD worse memory	Moderate
Foxe et al. ^39^	2024	Probable AD (NIA-AA/McKhann 2011)	Not specified	28 / 28	bvFTD	Memory / Executive	ACE-III, ROCF, TMT-A	AD worse memory	Moderate
Garcin et al. ^40^	2018	Probable AD (NIA-AA/McKhann 2011)	Cross-sectional	23 / 20	bvFTD	Executive	Not specified	Mixed results	Moderate
Gascón ^41^	2010	Probable AD (NINCDS-ADRDA)	Cross-sectional	25 / 9	bvFTD	Memory / Executive	DS-F	No difference	High
Giovagnoli et al. ^42^	2008	Probable AD (NINCDS-ADRDA)	Cross-sectional, Retrospective	77 / 40	bvFTD	Memory / Executive	ROCF, TMT-A, TMT-B	AD worse memory	Moderate
Gleichgerrcht et al. ^43^	2011	Probable AD (NINCDS-ADRDA)	Cross-sectional	25 / 25	bvFTD	Executive	TMT-B, WCST, FAS	Executive mixed	Moderate
Goodwin et al. ^16^	2025	Neuropathologically confirmed AD	Retrospective	1498 / 118	bvFTD	Memory / Executive	WMS, TMT-A, TMT-B	AD worse memory	Moderate
Grandi et al. ^44^	2025	Probable AD (NIA-AA/McKhann 2011)	Cross-sectional	45 / 33	bvFTD	Executive	FAB	No difference	Moderate
Gregory et al. ^45^	2002	Probable AD (NINCDS-ADRDA)	Cross-sectional	12 / 19	bvFTD	Memory / Social cognition	ROCF, DS-F, DS-B	AD worse memory	Moderate
Gressie et al. ^46^	2024	Probable AD (NIA-AA/McKhann 2011)	Cross-sectional	76 / 62	bvFTD	Social cognition	FAST	No difference	Moderate
Grossi et al. ^47^	2002	Probable AD (NINCDS-ADRDA)	Cross-sectional	11 / 14	bvFTD	Memory / Executive	ROCF, Stroop	AD worse memory	Moderate
Grossman et al. ^48^	2007	Neuropathologically confirmed AD	Cross-sectional, Retrospective	14 / 47	Not specified	Memory / Executive	TMT-B, DS-F, DS-B	AD worse memory	Moderate
Heidler-Gary et al. ^49^	2007	Probable AD (NINCDS-ADRDA)	Cross-sectional	30 / 25	svPPA, PNFA	Memory / Executive	RAVLT, TMT-A, TMT-B	AD worse memory	Moderate
Hodges et al. ^50^	1999	Probable AD (NINCDS-ADRDA)	Cross-sectional	9 / 9	bvFTD, svPPA	Memory / Executive	ROCF, WMS, DS-F	AD worse attention	Moderate
Hornberger et al. ^51^	2010	Probable AD (NINCDS-ADRDA)	Cross-sectional	15 / 11	bvFTD	Executive	HSCT, FAS	Executive mixed	Moderate
Hornberger et al. ^52^	2010	Probable AD (NINCDS-ADRDA)	Cross-sectional	64 / 39	bvFTD (progressors and phenocopy cases)	Memory / Executive	RAVLT, ROCF, WMS	Mixed results	Moderate
Jenner et al. ^53^	2006	Probable AD (NINCDS-ADRDA)	Cross-sectional	22 / 24	bvFTD	Memory / Executive	RAVLT, ROCF, TMT-B	AD worse memory	Moderate
Jiskoot et al. ^54^	2021	Probable AD (NIA-AA/McKhann 2011)	Cross-sectional, Retrospective	32 / 32	bvFTD	Social cognition	ERT	FTD worse social cognition	Moderate
Johnen et al. ^19^	2018	Biomarker-supported AD	Cross-sectional	27 / 31	bvFTD, svPPA, nfPPA	Social cognition / Executive	FAB, Mini-SEA, SEA	FTD worse social cognition	Moderate
Kamminga et al. ^55^	2014	Probable AD (NINCDS-ADRDA)	Cross-sectional	8 / 8	bvFTD, svPPA	Memory / Executive	RAVLT, ROCF, TMT-A	AD worse memory	Moderate
Kertesz et al. ^56^	2003	Probable AD (NINCDS-ADRDA)	Cross-sectional	52 / 52	Not specified	Memory / Language	WMS, WAB	AD worse memory	Moderate
Kiselica et al. ^17^	2021	Probable AD (NIA-AA/McKhann 2011)	Cross-sectional	1577 / 406	bvFTD	Executive	TMT-B, FAS	Executive mixed	Moderate
Kumfor et al. ^57^	2014	Probable AD (NINCDS-ADRDA)	Longitudinal	17 / 20	bvFTD	Social cognition	Ek-60F, TASIT	No difference	Moderate
Le Bouc et al. ^58^	2012	Probable AD (NIA-AA/McKhann 2011)	Cross-sectional	12 / 11	bvFTD	Social cognition	ToM	Executive mixed	Moderate
Lee et al. ^59^	2003	Probable AD (NINCDS-ADRDA)	Cross-sectional	10 / 12	bvFTD, svPPA	Memory / Executive	Category fluency	AD worse memory	Moderate
Leslie et al. ^60^	2016	Probable AD (NIA-AA/McKhann 2011)	Cross-sectional	14 / 14	bvFTD	Executive	TMT-B, DS-B, HSCT	Mixed results	Moderate
Libon et al. ^61^	2007	Probable AD (NINCDS-ADRDA)	Cross-sectional	38 / 71	SOC/EXEC (n = 16), svPPA (n = 18), PNFA (n = 13), CBD (n = 24)	Memory / Executive	TMT-B, DS-F, DS-B	AD worse memory	Moderate
Lindau et al. ^62^	2003	Probable AD (NINCDS-ADRDA)	Cross-sectional	16 / 19	Not specified	Memory / Executive	TMT-A, TMT-B, DS-F	Executive mixed	High
Luzzi et al. ^63^	2020	Biomarker-confirmed AD	Cross-sectional	25 / 24	bvFTD	Memory / Executive	RAVLT, ROCF, Stroop	AD worse memory	Moderate
Mariano et al. ^64^	2020	Probable AD (NIA-AA/McKhann 2011)	Cross-sectional	20 / 22	bvFTD	Memory / Social cognition	FMT, FAB, Mini-SEA	FTD worse social cognition	Moderate
Mariano et al. ^65^	2020	Biomarker-supported AD	Cross-sectional	25 / 27	bvFTD	Memory / Executive	FMT, DS-F, Stroop	AD worse memory	Moderate
Moura et al. ^15^	2021	Biomarker-supported AD	Cross-sectional	20 / 21	nondysexecutive-bvFTD, dysexecutive-bvFTD	Memory / Social cognition	FMT, DS-F, DS-B	FTD worse social cognition	Moderate
Nakaaki et al. ^66^	2007	Probable AD (NINCDS-ADRDA)	Cross-sectional	18 / 18	bvFTD	Executive	FAB	Mixed results	Moderate
Ossenkoppele et al. ^7^	2015	Biomarker-confirmed AD	Retrospective	133 / 59	bvFTD	Memory / Executive	RAVLT, CVLT, BFT	AD worse memory	Low
Pachana et al. ^67^	1996	Probable AD (NINCDS-ADRDA)	Retrospective	16 / 15	Not specified	Memory / Executive	RAVLT, ROCF, WMS	No difference	High
Panchal et al. ^68^	2016	Probable AD (NIA-AA/McKhann 2011)	Cross-sectional	18 / 15	bvFTD	Social cognition / Executive	WCST, FAS, D-KEFS	FTD worse social cognition	Moderate
Pennington et al. ^69^	2011	Probable AD (NIA-AA/McKhann 2011)	Cross-sectional	14 / 20	bvFTD	Memory	RAVLT, ROCF	No difference	Moderate
Perri et al. ^70^	2005	Probable AD (NINCDS-ADRDA)	Cross-sectional	39 / 19	bvFTD	Memory / Executive	ROCF, FAS, BNT	AD worse memory	Moderate
Perry and Hodges ^71^	2000	Probable AD (NINCDS-ADRDA)	Cross-sectional	10 / 15	temporal variant FTD (historical terminology; corresponding approximately to svPPA n = 5), bvFTD (frontal variant n = 10)	Memory / Executive	ROCF, WMS, WRMT	AD worse memory	Moderate
Possin et al. ^72^	2013	Probable AD (NINCDS-ADRDA)	Cross-sectional	26 / 22	bvFTD	Memory / Executive	FAS, Category fluency, SNQ	AD worse memory	Moderate
Pozueta et al. ^73^	2019	Biomarker-confirmed AD	Cross-sectional	32 / 55	bvFTD (n = 20), svPPA (n = 35)	Memory / Social cognition	ROCF, FCSRT, TMT-A	FTD worse social cognition	Moderate
Quaranta et al. ^74^	2012	Probable AD (NINCDS-ADRDA)	Cross-sectional	20 / 28	bvFTD	Memory / Executive	RAVLT, ROCF, Stroop	AD worse memory	Moderate
Ramanan et al. ^75^	2017	Biomarker-supported AD	Cross-sectional	29 / 44	bvFTD	Memory / Social cognition	DS-F, DS-B, Category fluency	FTD worse social cognition	Moderate
Ramanan et al. ^20^	2017	Probable AD (NIA-AA/McKhann 2011)	Longitudinal	33 / 31	bvFTD	Memory / Executive	ACE-R, RAVLT, ROCF	Executive mixed	Moderate
Ranasinghe et al. ^76^	2016	Probable AD (NINCDS-ADRDA)	Cross-sectional, Retrospective	674 / 204	bvFTD	Memory / Social cognition	CVLT, BFT, DS-F	AD worse memory	Moderate
Razani et al. ^77^	2001	Probable AD (NINCDS-ADRDA)	Cross-sectional	16 / 27	right-FTD, left-FTD	Memory / Executive	ROCF, Stroop, WCST	AD worse visuospatial	Moderate
Reul et al. ^4^	2017	Biomarker-confirmed AD	Cross-sectional, Retrospective	43 / 26	bvFTD	Memory / Social cognition	ROCF, WMS, VLMT	FTD worse social cognition	Moderate
Ritter et al. ^78^	2017	Neuropathologically confirmed AD	Cross-sectional, Retrospective	558 / 106	FTLD (bvFTD + two types of PPA)	Memory / Executive	TMT-A, TMT-B, DS-F	AD worse memory	Low
Salimi et al. ^79^	2019	Probable AD (NIA-AA/McKhann)	Cross-sectional	55 / 51	bvFTD	Memory / Executive	ACE-III, ACE-R, ROCF	AD worse memory	Moderate
Schubert et al. ^80^	2016	Probable AD (NIA-AA/International Consensus)	Longitudinal	31 / 22	bvFTD	Memory / Executive	ACE-R, ROCF, TMT-A	AD worse memory	Moderate
Siri et al. ^81^	2001	Probable AD (NINCDS-ADRDA)	Cross-sectional	14 / 14	Not specified	Memory / Executive	ROCF, WCST, BNT	AD worse memory	Moderate
Stopford et al. ^82^	2012	Probable AD	Not specified	38 / 26	bvFTD	Memory / Executive	DS-F, DS-B, Go/No-Go	AD worse memory	Moderate
Thompson et al. ^83^	2005	Probable AD (NINCDS-ADRDA)	Not specified	73 / 38	svPPA and PNFA excluded	Memory / Executive	DS-F, DS-B, FAS	FTD worse language	Moderate
Tu et al. ^84^	2017	Probable AD (NIA-AA/McKhann)	Cross-sectional	11 / 11	bvFTD	Memory / Executive	RAVLT, ROCF, DS-F	AD worse memory	Moderate
Wajman et al. ^85^	2019	Probable AD (NIA-AA 2011)	Cross-sectional	39 / 16	bvFTD	Executive	Category fluency	No difference	Moderate
Walker et al. ^86^	2005	Probable AD (NINCDS-ADRDA)	Cross-sectional	29 / 11	Not specified	Memory / Executive	RAVLT, ROCF, WMS	AD worse memory	Moderate
Wang et al. ^87^	2022	Probable AD (NIA-AA 2011)	Cross-sectional	26 / 22	bvFTD	Memory / Social cognition	AVLT, TMT-A, TMT-B	FTD worse social cognition	Moderate
Wicklund et al. ^88^	2004	Probable AD (NINCDS-ADRDA)	Cross-sectional	20 / 16	bvFTD, PPA	Memory / Language	WMS, TMT-A, BNT	AD worse memory	Moderate
Wicklund et al. ^89^	2007	Probable AD (NINCDS-ADRDA)	Longitudinal	84 / 66	bvFTD, PPA	Memory / Executive	TMT-A, TMT-B, FAS	AD worse memory	Moderate
Wilson et al. ^90^	2024	Probable AD (NIA-AA/McKhann 2011)	Cross-sectional	13 / 18	bvFTD	Memory / Executive	ACE-III, RAVLT, ROCF	AD worse memory	Moderate
Van den Berg et al. ^91^	2020	Probable AD (NINCDS-ADRDA)	Cross-sectional, Retrospective	55 / 85	bvFTD	Memory / Executive	RAVLT, VAT, RBMT	AD worse memory	Moderate
Van den Berg et al. ^92^	2022	Probable AD (NINCDS-ADRDA)	Cross-sectional	36 / 23	bvFTD	Social cognition / Executive	TMT-A, TMT-B, FAS	FTD worse social cognition	Moderate

ACE: Addenbrooke's Cognitive Examination; AD: Alzheimer's disease; AVLT: Auditory Verbal Learning Test; BFT: Benson Figure Test; BNT: Boston Naming Test; bvFTD: behavioral variant frontotemporal dementia; BVMT-R: Brief Visuospatial Memory Test-Revised; CBD: corticobasal degeneration; CVLT: California Verbal Learning Test; D-KEFS: Delis-Kaplan Executive Function System; DS-B: Digit Span Backward; DS-F: Digit Span Forward; eAD: early-onset AD; Ek-60F: Ekman 60-Faces Test; ERT: Emotion Recognition Task; FAB: Frontal Assessment Battery; FAST: Facial Affect Selection Task; FCSRT: Free and Cued Selective Reminding Test; FMT: Figure Memory Test; FTD: frontotemporal dementia; FTLD: frontotemporal lobar degeneration; HSCT: Hayling Sentence Completion Test; HVLT-R: Hopkins Verbal Learning Test-Revised; IFS: INECO Frontal Screening; lvFTD: language variant FTD; Mini-SEA: Mini Social Cognition and Emotional Assessment; nfPPA; non-fluent/agrammatic variant PPA; NIA-AA: National Institute on Aging and Alzheimer's Association; NINCDS-ADRDA: National Institute of Neurological and Communicative Disorders and Stroke and the Alzheimer's Disease and Related Disorders Association; PNFA: progressive non-fluent aphasia; PPA: primary progressive aphasia; RAVLT: Rey Auditory Verbal Learning Test; RBMT: Rivermead Behavioural Memory Test; ROCF: Rey–Osterrieth Complex Figure Test; SEA: Social Cognition and Emotional Assessment; SET: Story-based Empathy Task; SNQ: Social Norms Questionnaire; svPPA: semantic variant primary progressive aphasia; TASIT: The Awareness of Social Inference Test; TMT: Trail Making Test; ToM: Theory of Mind; VAT: Visual Association Test; VLMT: Verbal Learning and Memory Test; WAB: Western Aphasia Battery; WCST: Wisconsin Card Sorting Test; WMS: Wechsler Memory Scale; WRMT: Woodcock Reading Mastery Test.

Due to substantial heterogeneity in study design, patient populations and neuropsychological assessment methods, a meta-analysis was not performed. Instead, the findings were summarized narratively and organized according to cognitive domains.

Given the heterogeneity of neuropsychological study designs, a qualitative risk-of-bias assessment adapted for observational neuropsychological studies was performed. Studies were evaluated based on diagnostic clarity, sample size, group comparability, standardization of the neuropsychological assessment, consideration of confounding factors, and the appropriateness of the statistical analyses. The overall risk of bias was categorized as low, moderate or high. The assessment was performed by the review team and discrepancies were resolved by discussion.

## Results

### Study characteristics

A total of 80 studies met the inclusion criteria and were included in the review. The characteristics of the studies are summarized in Table 2. In several studies, disease stage was not explicitly reported. However, comparability between patient groups was often achieved through matching for Mini-Mental State Examination (MMSE) or Clinical Dementia Rating (CDR) scores, or by adjusting for disease severity in the analyses. Across most studies, AD and FTD groups showed broadly comparable global cognitive impairment and disease severity. Only two studies reported significant differences in disease stage between groups.

**Table 2.** Characteristics of included studies.

Characteristic	Summary
Number of studies	80
Study design	Predominantly cross-sectional studies (n = 73), with a smaller number of longitudinal (n = 4) and retrospective studies (n = 3)
Sample size	Highly variable across studies (AD: 8-1577 / FTD: 8-406)
Diagnostic groups	AD versus FTD
FTD subtypes	Mainly bvFTD; some studies included PPA
Disease stage	Often not explicitly reported; typically matched using MMSE or CDR
Neuropsychological domains assessed	Memory, social cognition, executive functions, language, attention, visuospatial abilities
Commonly used tests	RAVLT, ROCF, WMS, Mini-SEA, Faux-Pas Test, TMT-A/B, Stroop, VOSP

AD: Alzheimer's disease; bvFTD: behavioral variant frontotemporal dementia; CDR: Clinical Dementia Rating; FTD: frontotemporal dementia; Mini-SEA: Mini Social Cognition and Emotional Assessment; MMSE: Mini-Mental State Examination; PPA: primary progressive aphasia; RAVLT: Rey Auditory Verbal Learning Test; ROCF: Rey–Osterrieth Complex Figure Test; TMT: Trail Making Test; VOSP: Visual Object and Space Perception; WMS: Wechsler Memory Scale.

### Neuropsychological domains

The findings were analyzed according to six neuropsychological domains: memory, social cognition, executive functions, language, attention and visuospatial functions. A domain-based summary of the results is provided in Table 3.

**Table 3.** Neuropsychological domains differentiating ad and FTD.

Domain	Direction of impairment	Consistency	Example tests	Interpretation
Episodic memory	AD > FTD impairment	High	RAVLT, ROCF (delayed recall), WMS	Most consistently reported marker of AD
Social cognition	FTD > AD impairment	High	Mini-SEA, Faux-Pas, Ekman-60	Most consistently reported marker of FTD
Executive functions	Mixed	Low–moderate	TMT-B, Stroop, FAS, HSCT	Subdomain-dependent differentiation
Language	Mixed / subtype-dependent	Low	BNT, naming tests	Relevant mainly in PPA variants
Attention	Greater impairment in AD than FTD	Low	TMT-A, DSST	Supportive marker only
Visuospatial functions	Greater impairment in AD than FTD	Moderate	ROCF (copy), VOSP	Secondary marker for AD

AD: Alzheimer's disease; BNT: Boston Naming Test; DSST: Digit Symbol Substitution Test; FTD: frontotemporal dementia; HSCT: Hayling Sentence Completion Test; Mini-SEA: Mini Social Cognition and Emotional Assessment; PPA: primary progressive aphasia; RAVLT: Rey Auditory Verbal Learning Test; ROCF: Rey–Osterrieth Complex Figure Test; TMT: Trail Making Test; VOSP: Visual Object and Space Perception; WMS: Wechsler Memory Scale.

### Memory

Across most included studies, particularly those using delayed recall paradigms, memory was identified as one of the most reliable domains for distinguishing AD from FTD.^4,7,20,25,27,51,80,91^ Patients with AD consistently performed worse than patients with FTD in memory tasks, particularly those involving episodic memory. This pattern was most evident in delayed recall measures, such as the Rey Auditory Verbal Learning Test (RAVLT), the Rey–Osterrieth Complex Figure Test (ROCF) and the Wechsler Memory Scale (WMS). Several studies further suggested that the differentiation between AD and FTD depended not only on overall memory performance, but also on the specific memory process assessed, including encoding, delayed recall and recognition memory.^7,27,91^ In particular, impairments in encoding and delayed recall appeared to be more characteristic of AD, whereas recognition performance was often relatively less affected in FTD. Overall, episodic memory impairment appeared to be a characteristic feature of AD, whereas memory performance in FTD was generally less severely affected and often relatively preserved in the early stages of the disease.

### Social cognition

Social cognition emerged as one of the most consistently discriminative markers of FTD across studies.^14,15,18,29,33,36,54,64,75,87,90,92^ Across the large majority of included studies, patients with FTD demonstrated more pronounced deficits than those with AD, particularly in tasks evaluating theory of mind and emotion recognition. Frequently used discriminative measures included the Mini Social Cognition and Emotional Assessment (Mini-SEA), the Faux-Pas Test, the Facial Emotion Recognition Test (FERT) and the Ekman 60 Faces Test. These deficits were often already evident in the early stages of the disease, which supports the relevance of social cognition for the differential diagnosis of FTD.

### Executive functions

Findings for executive functions were markedly heterogeneous, with only some executive subdomains consistently differentiating between AD and FTD.^13,17,24,30,43,51,60,61,72,82^ Some executive measures differentiated FTD from AD, whereas others did not. Patients with FTD tended to perform worse in tasks related to phonemic fluency and inhibition, such as phonemic verbal fluency tasks (e.g., FAS) and the Hayling Sentence Completion Test (HSCT). In contrast, patients with AD often performed worse on the Trail Making Test Part B (TMT-B), particularly in more advanced stages of the disease. Other commonly used measures, including Digit Span, the Stroop Test and the Wisconsin Card Sorting Test (WCST), produced inconsistent results across studies. Overall, the discriminative value of executive functioning depended strongly on the specific subcomponent of executive function assessed and on disease stage.

### Language

Language showed limited overall value for differentiating between AD and FTD.^35,49,50,56,63,71,73,83,88^ In most studies, no clear differences between groups were observed. If significant differences were reported, these would often be driven by specific FTD subtypes, particularly semantic dementia or primary progressive aphasia. Therefore, language did not emerge as a robust general marker for distinguishing AD from FTD, but it may be useful in the context of specific subtypes.

### Attention

Attention showed only limited and inconsistent discriminative value.^48,62,75,78,82^ Some studies suggested that patients with AD performed worse than patients with FTD on the Trail Making Test Part A (TMT-A) and the Digit Symbol Substitution Test (DSST). However, this pattern was less consistent than that observed for memory or social cognition. Overall, attention appeared to provide supplementary, rather than standalone, diagnostic value.

### Visuospatial functions

Visuospatial functions also contributed to differential diagnosis, whereby patients with AD generally showed poorer performance than those with FTD.^39,47,77,79,84^ This was particularly evident in visuoconstructional and visuoperceptual tasks such as the Rey-Osterrieth Complex Figure (ROCF) copy, the Benson Figure copy and the Visual Object and Space Perception (VOSP) Battery. However, compared with memory, visuospatial functions appeared to be less consistently discriminative, and should therefore be regarded as supportive rather than primary markers of AD.

### Screening instruments

In addition to domain-specific neuropsychological tests, some studies used global cognitive screening instruments, such as variants of the Addenbrooke's Cognitive Examination (ACE), the Edinburgh Cognitive and Behavioural ALS Screen (ECAS), the Frontal Assessment Battery (FAB) and the INECO Frontal Screening (IFS).^22,24,31,32,34,44,60,81^ As these instruments assess multiple domains simultaneously, they were of limited value for domain-specific interpretation and did not enable consistent differentiation between AD and FTD.

### Overall pattern

Taken together, the results showed a clear domain-specific pattern. Overall, episodic memory emerged as the most consistently reported neuropsychological marker of AD, whereas social cognition emerged as the most consistently reported marker of FTD. Executive functions, language, visuospatial abilities, attention, and behavioral measures provided additional discriminatory value depending on the clinical phenotype and the specific tests applied (Figure 2).

**Figure 2.**
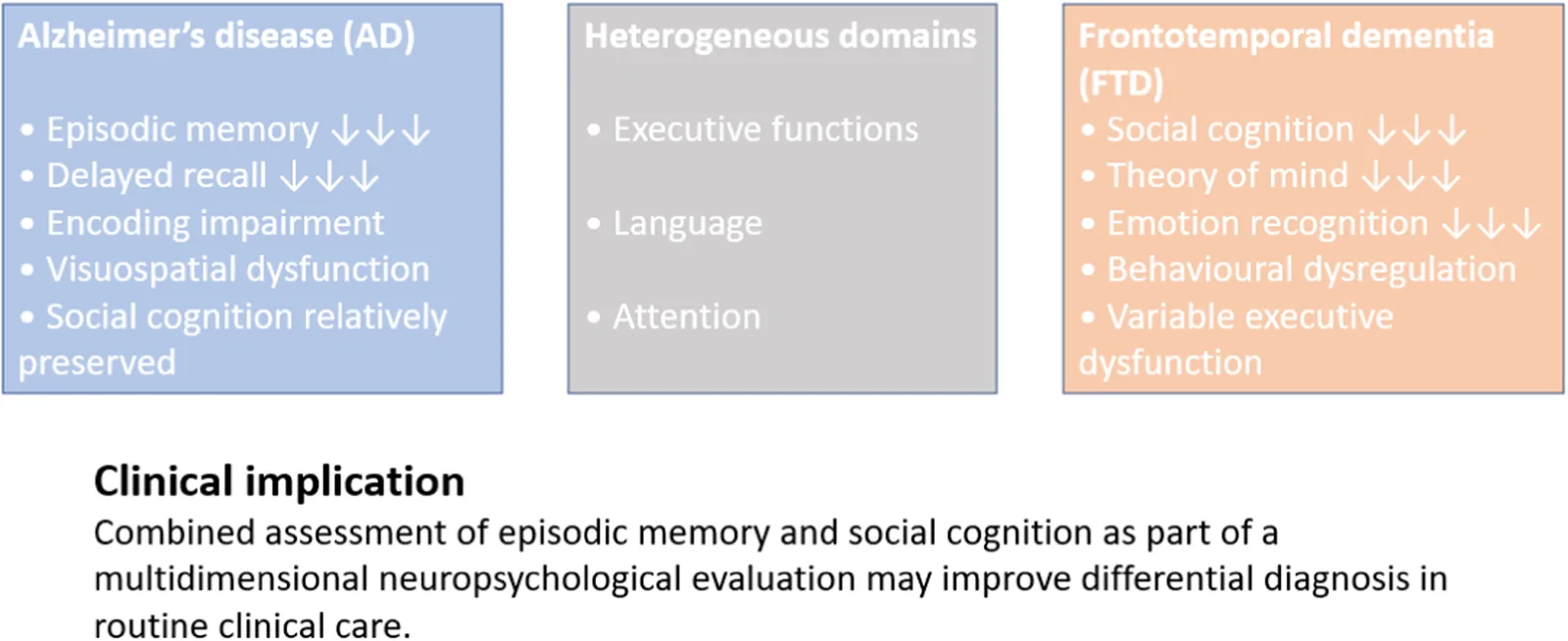
Domain-specific neuropsychological patterns differentiating Alzheimer's disease and frontotemporal dementia.

## Discussion

This systematic review aimed to identify the neuropsychological domains and testing procedures that can most reliably differentiate between AD and FTD. Across the included studies, episodic memory emerged as the most consistently reported discriminator between AD and FTD. However, this finding predominantly reflects studies including patients with typical amnestic AD and may not fully apply to atypical AD phenotypes. Overall, the included studies demonstrated a clear domain-specific pattern. Episodic memory and social cognition were the most consistently reported discriminating domains, whereas executive functions, language, attention, and visuospatial abilities contributed substantially depending on the clinical phenotype and the specific neuropsychological tests employed.

The strong discriminative value of episodic memory is consistent with the characteristic involvement of medial temporal lobe structures in AD.^1,10,15^ Across studies, delayed recall measures were particularly effective at distinguishing AD from FTD, which supports their central role in clinical assessment.^4,20,25,27,51,80,91^ This is consistent with established models of AD pathology, which emphasize early hippocampal dysfunction and impaired encoding processes.^3^ Moreover, several studies suggested that differences between AD and FTD may not only concern the severity of memory impairment, but also the underlying memory processes involved, particularly encoding, delayed recall and recognition performance.^7,27,91^ This may reflect distinct neuroanatomical and cognitive mechanisms underlying memory dysfunction in the two disorders. Conversely, deficits in social cognition, including theory of mind and emotion recognition, consistently differentiated FTD from AD and were often detectable in the early stages of the disease.^14,15,18,29,33,36,54,64,75,87,90,92,93^ These findings are consistent with previous research showing that deficits in social cognition are a core feature of bvFTD and may precede other cognitive impairments.^14,15,93^ Beyond its role in differential diagnosis, social cognition has important implications for everyday functioning, interpersonal relationships, caregiver burden, behavioral symptoms, and decision-making capacity. Moreover, impairments in social cognition are not unique to FTD but have also been described across a range of neurodegenerative disorders, including AD and other conditions. Therefore, assessment of social cognition may provide clinically relevant information that extends beyond diagnostic discrimination alone.

In contrast, executive functions showed substantial heterogeneity across studies.^13,17,24,30,43,60,61,72,82^ While some subcomponents, such as phonemic fluency and inhibition, tended to be more impaired in FTD, other measures, including set-shifting tasks, did not consistently differentiate between groups, or showed greater impairment in AD at more advanced stages of the disease. This variability likely reflects the multidimensional nature of executive functioning and the involvement of partially overlapping neural networks.^13^ Similarly, language did not emerge as a reliable general marker for differentiation, although it discriminated from specific FTD subtypes, particularly in studies focusing on language-predominant variants.^35,49,50,56,63,71,73,83,88^

Several methodological factors likely contributed to the heterogeneity observed across the included studies. An additional source of heterogeneity relates to the evolution of diagnostic criteria over time. No restrictions regarding publication year were applied in order to provide a comprehensive overview of the available literature. Consequently, several included studies predated the revised international diagnostic criteria for bvFTD proposed by Rascovsky et al.^12^ and the consensus criteria for primary progressive aphasia variants proposed by Gorno-Tempini et al..^94^ Diagnostic criteria for both FTD and AD have evolved considerably across the study period, which may have influenced patient classification and comparability between studies. Furthermore, diagnostic certainty for AD varied across studies, ranging from clinical diagnoses based on NINCDS-ADRDA or NIA-AA criteria to biomarker-supported, biomarker-confirmed, and neuropathologically confirmed cohorts. In addition, while the overwhelming majority of included studies investigated patients with AD dementia, only a small number included individuals with prodromal or mild AD. Likewise, most studies focused on the typical amnestic presentation of AD, whereas atypical phenotypes such as behavioral/dysexecutive AD, posterior cortical atrophy, or logopenic primary progressive aphasia were only rarely represented. Consequently, the findings of this review primarily reflect the neuropsychological profile of typical AD and should not be generalized to the full clinical spectrum of AD. Therefore, separate qualitative synthesis according to disease stage was considered but was not performed because of the limited number and substantial heterogeneity of studies involving prodromal AD. Accordingly, the findings of this review should be interpreted within the historical diagnostic context of the individual publications. Firstly, the inclusion of different FTD phenotypes contributed to heterogeneity across studies, particularly in the language domain. While bvFTD represented the largest subgroup, several studies also included language variants or atypical parkinsonian syndromes. Studies explicitly including semantic or language variants reported more pronounced language differences compared with studies restricted to bv FTD. Disease stage and comparability between study groups also played an important role. While memory deficits are typically evident early in AD, impairments in other domains, such as executive functions or visuospatial abilities, may emerge later in the disease course.^10^ Finally, variability in the neuropsychological tests limited comparability between studies and likely contributed to inconsistent findings, particularly with regard to executive functions. Such heterogeneity is a well-recognized challenge in neuropsychological research and complicates direct comparisons between studies.^13^

In addition, the included studies span more than two decades, during which both FTD and AD diagnostic criteria have undergone substantial revisions. This temporal heterogeneity may have influenced diagnostic accuracy and comparability between studies. Another important limitation of the included studies is their frequent use of relatively small sample sizes. Small samples reduce statistical power and may lead to inconsistent or non-replicable findings, particularly in a clinically heterogeneous condition such as FTD. Furthermore, the heterogeneity of study designs, patient populations and test procedures prevented a quantitative meta-analysis. Consequently, the results of this review are based on a narrative synthesis and should be interpreted with this in mind. Another limitation is that only PubMed was searched. Although PubMed indexes the majority of biomedical literature relevant to this topic, studies indexed exclusively in other databases may have been missed. An additional limitation is that study selection and data extraction were performed by a single reviewer. Although predefined eligibility criteria and standardized data extraction procedures were applied, this approach may have increased the risk of selection bias and data extraction errors. Although a simplified risk-of-bias assessment was performed, the appraisal was qualitative and not based on a fully independent duplicate rating procedure.

Despite these limitations, the findings of this review have important implications for clinical practice. The results suggest that neuropsychological assessment should incorporate a multidimensional approach^10,14,15,17,20^ rather than relying solely on memory-based measures. In particular, combining episodic memory assessment, especially tasks evaluating delayed recall and encoding processes, with measures of social cognition, including theory of mind and emotion recognition, appears to be a robust and clinically applicable strategy for differentiating AD from FTD. One particularly relevant implication for clinical practice is that memory-focused screening approaches alone may not adequately capture the characteristic cognitive profile of bvFTD, particularly in the early stages of the disease. In patients presenting with behavioral changes, impaired interpersonal functioning, or psychiatric symptoms, despite having relatively preserved episodic memory, assessing social cognition in addition to conventional neuropsychological testing can substantially improve diagnostic accuracy. Therefore, measures targeting theory of mind and emotion recognition, such as the Mini-SEA or Faux-Pas paradigms, appear to be a promising addition to conventional neuropsychological batteries. Furthermore, despite the increasing availability of fluid and imaging biomarkers, neuropsychological assessment remains highly relevant for the clinical evaluation of patients with suspected neurodegenerative disorders, particularly in cases of atypical presentation or in settings where access to biomarkers is limited. Consequently, assessment of episodic memory and social cognition should form part of a comprehensive multidimensional neuropsychological evaluation rather than being interpreted in isolation. Additional assessment of executive, language, visuospatial, attentional, and behavioral domains may further improve diagnostic accuracy depending on the suspected clinical phenotype.^3^

Future research should focus on larger, well-characterized cohorts with a clear FTD subtype classification. Longitudinal study designs are needed to better understand how cognitive profiles evolve across disease stages, and to determine the stability of domain-specific markers over time. Furthermore, developing and validating standardized neuropsychological test batteries integrating multiple cognitive domains, particularly social cognition, could improve diagnostic accuracy and facilitate clinical implementation. Future studies may also investigate whether more specific assessments of social cognition, insight or behavioral regulation further improve differential diagnosis between AD and FTD.^95–98^ Across the included studies, episodic memory and social cognition emerged as the most consistently reported neuropsychological domains distinguishing AD from FTD. However, executive, language, visuospatial, attentional, and behavioral functions also contributed substantially to the differential diagnosis, particularly in specific clinical phenotypes. Therefore, neuropsychological differentiation should be conceptualized as an integrated multidimensional profile incorporating cognitive, behavioral, and functional domains rather than relying on any single domain or combination of only two domains. Consequently, the findings of this review primarily reflect the neuropsychological profile of typical AD and should not be generalized to the full clinical spectrum of AD.
